# 侵袭性NK细胞白血病22例临床特征和预后分析

**DOI:** 10.3760/cma.j.issn.0253-2727.2022.05.013

**Published:** 2022-05

**Authors:** 文斯 钱, 琳 沈, 乌丽盼·甫拉提, 敏 吴, 洁娴 马, 萍萍 陈, 瑜 徐, 彦晖 谢

**Affiliations:** 复旦大学附属华东医院血液科，上海 200040 Department of Hematology, Huadong Hospital, Fudan University, Shanghai 200040, China

侵袭性NK细胞白血病（aggressive NK cell leukemia,ANKL）是一种罕见且极具侵袭性的NK细胞恶性肿瘤，发病率占淋巴造血系统肿瘤的0.1％左右[1]。ANKL具有早期诊断困难、发病率极低、临床进程快、治疗效果差及病死率高等特点。关于ANKL的临床研究较少，大部分为病例报道。本研究回顾性分析了22例ANKL患者的临床特征及预后特点，为临床提供治疗思路。

## 病例与方法

1. 病例资料：共收集2015年2月至2020年12月本院收治的病理学诊断明确的22例ANKL的患者。所有患者均按照淋巴瘤诊治常规进行血液学和影像学等检查，病理类型依据WHO淋巴造血系统肿瘤分类2008版[2]进行分组，疗效评估采用2014年Lugano标准[3]。

2. 临床数据：本研究收集的临床数据主要包括患者性别、年龄、临床表现、Ann Arbor临床分期、初诊时HGB、WBC、中性粒细胞绝对计数（ANC）、PLT、白蛋白（Alb）、乳酸脱氢酶（LDH）、β_2_微球蛋白（β_2_-MG）、碱性磷酸酶（AKP）、血肌酐（Scr）、血浆EBV-DNA水平以及治疗方式等。

3. 随访：随访截至2021年5月1日，采取住院复查、门诊复查、电话随访等方式，全部患者均获得随访。总生存（OS）时间定义为疾病确诊日期至患者死于任何疾病、末次随诊或截止观察日期的时间。

4. 统计学处理：采用SPSS 22软件进行统计分析，计数资料以例（％）表示，计量资料以中位数（范围）表示。生存率计算用Kaplan-Meier法，组间生存率的比较应用Log-rank检验，对于预后评价的多因素分析采用Cox回归方法，采用GraphPad-prism 7绘制生存曲线。*P*<0.05为差异有统计学意义。

## 结果

1. 临床特征：22例ANKL患者中位发病年龄为35（15～70）岁，年龄≥60岁4例（18.2％），<60岁18例（81.8％）；男16例（72.7％），女6例（27.3％），男女比例为8∶3；18例（81.8％）伴有噬血细胞综合征，21例（95.4％）初诊时有发热；15例（68.2％）初诊时一般体能状态较差，美国东部肿瘤协作组（ECOG）评分>2分；有19例患者进行血清EBV-DNA检测，其中18例（81.8％）阳性；ANKL免疫表型通过流式细胞术分析，细胞不表达胞膜抗原CD3（100％，0/22），但表达胞质抗原CD3（40.9％，9/22）、CD56（100％，22/22）、CD16（31.8％，7/22）、CD2（90.9％，20/22）、CD7（40.9％，9/22）。

2. 实验室检查：22例ANKL患者中位 WBC 3.3（0.2～12.2）×10^9^/L，中位HGB 98（60～130）g/L，中位ANC 1.70（0.15～10.71）×10^9^/L，中位PLT 39（6～263）×10^9^/L。生化常规检查中，中位Alb 31.5（21.9～40.0）g/L，中位Scr 65.2（28.3～321.1）mmol/L，中位AKP 180（25～1124）U/L，中位β_2_-MG 4.36（2.00～24.47）mg/L，中位LDH 550.9（128.8～2537.2）U/L。

3. 总体治疗及生存情况：22例ANKL患者在初诊时接受培门冬酶联合GDP方案（吉西他滨+顺铂+地塞米松）或者GEMOX方案（吉西他滨+奥沙利铂）治疗，对于合并噬血细胞综合征的患者在化疗前采用HLH-1994方案[4]控制相应症状。治疗1个疗程后40.9％（9/22）患者治疗有效，无患者获得完全缓解，诱导治疗后有2例患者接受自体造血干细胞移植，3例患者接受异基因造血干细胞移植。

随访至2021年5月1日，中位随访24.33个月，14例患者死亡，中位OS时间为4.03个月，2年的OS率为32.468％（图1）。

**图1:**
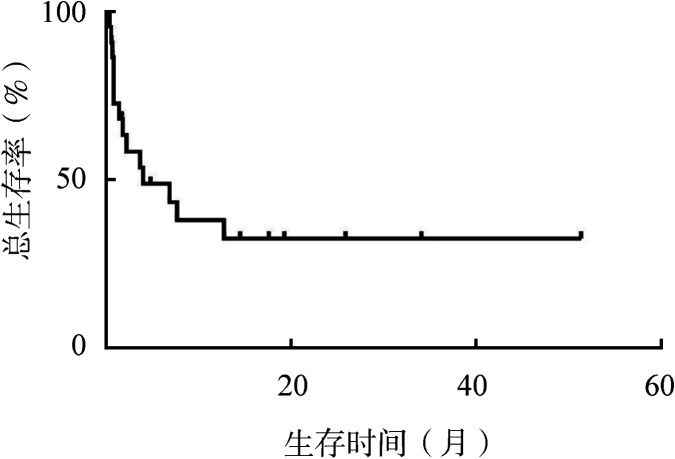
侵袭性NK细胞白血病患者的总生存曲线

4. 生存预后分析：单因素分析结果显示，性别、ECOG评分、外周血HGB水平、AKP水平、是否进行造血干细胞移植影响ANKL患者的OS（表1）。22例ANKL患者中，男性患者组的中位OS时间为7.67个月，女性患者组的中位OS时间为0.83个月，差异具有统计学意义（*P*＝0.018）。ECOG评分≤2分组患者的中位OS时间未达到，ECOG评分>2分组患者的中位OS时间为2.20个月，差异具有统计学意义（*P*＝0.029）。HGB≤90 g/L组患者的中位OS时间为1.80个月，HGB>90 g/L患者的中位OS时间未达到，差异具有统计学意义（*P*＝0.004）。AKP≥180 U/L组患者的中位OS时间为1.40个月，AKP<180 U/L患者的中位OS时间未达到，差异具有统计学意义（*P*＝0.001）。进行造血干细胞移植组患者的中位OS时间未达到，未进行造血干细胞移植组患者的中位OS时间为2.20个月，差异具有统计学意义（*P*＝0.016）。

**表1:** 影响侵袭性NK细胞白血病总生存的单因素分析结果

因素	*χ^2^*值	*HR*（95％ *CI*）	*P*值
年龄（<60岁，≥60岁）	0.080	0.831（0.230~3.003）	0.777
性别（男，女）	5.637	3.432（1.167~10.093）	0.018
噬血细胞综合征（有，无）	1.284	0.326（0.043~2.506）	0.257
ECOG评分（≤2分，>2分）	4.770	4.651（1.027~21.057）	0.029
WBC（≥3.0×10^9^/L，<3.0×10^9^/L）	0.174	1.253（0.433~3.625）	0.677
HGB（≥90 g/L，<90 g/L）	8.406	5.121（1.523~17.219）	0.004
ANC（≥2.0×10^9^/L，<2.0×10^9^/L）	0.044	1.120（0.387~3.237）	0.834
PLT（≥50×10^9^/L，<50×10^9^/L）	0.067	0.869（0.301~42.513）	0.796
Alb（≥30 g/L，<30 g/L）	1.285	1.838（0.632~5.347）	0.257
β_2_-MG（≥5 mg/L，<5 mg/L）	0.940	0.598（0.209~1.710）	0.332
AKP（≥180 U/L，<180 U/L）	11.951	0.124（0.032~0.479）	0.001
Scr（≥70 mmol/L，<70 mmol/L）	0.152	0.811（0.283~2.328）	0.697
LDH（≥500 U/L，<500 U/L）	0.041	1.115（0.390~3.189）	0.839
造血干细胞移植（是，否）	5.838	1.022（1.001~1.043）	0.016

注：ECOG：美国东部肿瘤协作组；ANC：中性粒细胞绝对计数；Alb：白蛋白；β_2_-MG：β_2_微球蛋白；AKP：碱性磷酸酶；Scr：血肌酐；LDH：乳酸脱氢酶

## 讨论

2008年WHO更新的造血及淋巴组织肿瘤分类[2]中，将ANKL正式纳入成熟T/NK细胞组织肿瘤的范畴。ANKL是一种罕见且高度侵袭性的恶性肿瘤，临床预后差，高发于亚洲人，任何年龄都可以发病，主要发生于少年和青壮年，患者的中位发病年龄42岁左右[5]–[7]，男女比例接近2∶1[8]。流式细胞术免疫表型分析是诊断ANKL的主要手段，通常ANKL的典型免疫表型为CD2^+^、sCD3^−^、cCD3^+^、CD56^+^、CDl6^+^，但可以有CD7、CD8等其他T细胞标志表达[9]。本研究纳入22例ANKL患者，中位发病年龄为35（15～70）岁，男女比例为8∶3。22例ANKL患者的免疫表型为sCD3^−^100％，cCD3^+^40.9％，CD56^+^100％，CD16^+^31.8％，CD2^+^90.9％，CD7^+^40.9％。

ANKL患者通常伴有发热、肝脾肿大、全血细胞减少等全身症状，多并发噬血细胞综合征，引起多器官功能衰竭和弥散性血管内凝血[9]。本组患者发病时21例（95.5％）出现发热，18例（81.8％）伴有噬血细胞综合征。大多数ANKL病例的诊断是根据外周血、骨髓或组织中存在NK肿瘤细胞，形态学上，有些患者有典型的大颗粒淋巴细胞特征[10]。ANKL表现为多器官受累的全身性疾病，Ishida等[11]分析34例ANKL患者，其中有19例患者发病时伴有噬血。从实验室检查方面，本研究中ANKL患者初诊时多伴有血细胞和Alb水平的减低，且LDH、AKP以及β_2_-MG水平的升高，提示着疾病的消耗及肿瘤负荷较大。

本研究中22例ANKL患者的中位OS时间达4.03个月，2年的OS率为32.5％。Tang等[8]进行一项多中心研究，回顾性分析113例ANKL患者，中位OS时间将近55 d，1年OS率为4.42％；Suzuki等[6]分析22例ANKL患者，中位OS时间为58 d；Ishida等[11]回顾性分析多中心的34例ANKL患者，中位OS时间为51 d。大部分国内外研究ANKL患者的中位OS时间为2个月左右。本组患者的生存较好，一方面可能与部分患者进行了造血干细胞移植相关，预后分析中进行造血干细胞移植患者的中位OS时间直至随访结束未达到，而未进行造血干细胞移植患者仅为3.3个月；另一方面可能与噬血细胞综合征的有效控制有关。

对ANKL患者的预后进行单因素分析，发现患者的性别、ECOG评分、外周血HGB水平、AKP水平、是否进行造血干细胞移植影响患者OS。Suzuki等[12]的研究同样显示患者的体能状态影响患者的预后。大多数研究表明移植是患者预后的影响因素，Tang等[8]发现接受造血干细胞移植患者的中位OS时间可达300 d，2年OS率为42.85％；Hussein等[13]发现接受造血干细胞移植的ANKL患者OS期可达1年以上，未接受移植的患者中位OS时间仅2个月。

血清AKP水平常常反映肿瘤的负荷，AKP水平越高，ANKL患者的肿瘤负荷越重，疾病进程越快，预后越差。噬血细胞综合征是免疫系统异常活化，产生大量细胞因子引起的一系列综合征[14]，发病极其凶险，Han等[15]报道淋巴瘤合并噬血细胞综合征往往提示预后较差，早期死亡的风险提高。另外本中心回顾性分析淋巴瘤相关的噬血细胞综合征的病例，患者的中位OS时间可达10.2个月，与我们早期诊断以及在诊断未明时对噬血细胞综合征的积极控制有关[16]。本研究中ANKL患者合并噬血细胞综合征与否中位OS时间差异无统计学意义，可能与不伴有噬血细胞综合征的患者（4例）较少有关。

ANKL患者的治疗，目前尚无确切的治疗方案。多数患者对传统的CHOP和CHOP样方案反应较差，目前认为比较有效的是以左旋门冬酰胺酶为基础的化疗方案，联合造血干细胞移植[17]–[18]。夏晶等[19]观察5例ANKL患者化疗后接受异基因造血干细胞移植，中位随访 23（2～69）个月，3例存活，2例死于复发。Fujimoto等[20]分析59例接受异基因造血干细胞移植的ANKL患者，发现31例诱导治疗无效的患者在接受异基因造血干细胞移植后，15例（48％）获得完全缓解，且移植后12个月仍有29％（17/59）的患者存活，而这些患者的5年OS率高达85.2％，表明异基因造血干细胞移植可以使部分ANKL患者获得长期缓解。

综上所述，ANKL十分罕见且临床预后较差，疾病发展迅速，多伴有噬血细胞综合征，目前没有确切的治疗方案。
